# Special Issue “Early Intervention for Hearing Loss in Children: Drafting from Theory to Clinical Practice”

**DOI:** 10.3390/jcm11113166

**Published:** 2022-06-02

**Authors:** Daniel Holzinger, Johannes Fellinger, Johannes Hofer

**Affiliations:** 1 Research Institute for Developmental Medicine, Johannes Kepler University Linz, 4020 Linz, Austria; johannes.fellinger@bblinz.at (J.F.); johannes.hofer@bblinz.at (J.H.); 2 Institute of Neurology of Senses and Language, Hospital of St. John of God, 4020 Linz, Austria; 3 Institute of Linguistics, University of Graz, 8010 Graz, Austria; 4 Division of Social Psychiatry, University Clinic for Psychiatry and Psychotherapy, Medical University of Vienna, 1090 Vienna, Austria; 5 Department of Paediatrics I, Innsbruck Medical University, 6020 Innsbruck, Austria

Globally, around 34 million children are affected by disabling hearing loss. Among Western newborns hearing loss shows a prevalence of about 2 per thousand, with about 4 times higher rates in other parts of the world such as Sub-Saharan Africa or South Asia. The scene of childhood hearing loss diagnosis and intervention has rapidly changed within the past two decades. Newborn hearing screening, followed by timely diagnosis and fitting with hearing technology, combined with early access to family-centered early intervention programs has shown significant effects on child language and psycho-social development and on family quality of life. Childhood hearing loss has thus proved to be a condition with highly malleable outcomes. The effectiveness of early intervention is largely determined by the quality of national and regional multidisciplinary networks and the active involvement of families.

An overarching model of early intervention is presented by our group [1]. Quality and quantity of parent-child interaction is shown to be a critical working mechanism that effects child language and social communication development, and consequently learning and psycho-social outcomes. Multidimensional intervention integrating family-centered early intervention and multidisciplinary clinical services can effectively support parent-child interaction by supporting families to cope with the manifold stressors following the diagnosis of their child’s hearing loss and in their everyday implementation of well adapted communication strategies. In addition to early identification and diagnosis, specialized clinical services provide a medical home that guarantees well fitted hearing devices, developmental surveillance to guide intervention and treatment and counselling particularly in case of additional disabilities.

Zee and Dirks [2] demonstrate the high diversity of child and family characteristics of children with hearing loss family-centered intervention is required to adapt to. The rate of additional disabilities and the degree of family involvement are shown to predict language outcomes.

Two studies refer to the early identification of hearing loss. Elliot et al. [3] investigate parental support needs on their way from newborn hearing screening to enrolment in early intervention services. Their findings demonstrate the need of parents for balanced information after their baby failed newborn hearing screening, opportunities for mental health support and the inclusion of both parents in the support. The study on newborn hearing screening and intervention in children with unilateral hearing loss in Nordic countries by Laugen et al. [4] reveals large within country variations in organizing health care and clinical decision making that can delay timely diagnosis and intervention. Therefore, mandatory guidelines are warranted.

The retrospective chart analysis by Minami et al. [5] examines the results of newborn hearing screening in individuals with congenital cytomegalovirus (cCMV) associated hearing loss and demonstrates high risk of missing this condition through newborn hearing screening. Consequently universal cCMV screening is recommended. In an overall small sample of symptomatic cCMV patients under valganciclovir treatment diagnosed as early as 3 weeks after birth Kido et al. [6] prospectively describe no difference in the changes of blood and urine viral loads between infants with and without hearing loss at 6 months of corrected age and at six-weeks and 6-months post treatment. These results add to the scarce literature on changes in viral load during valganciclovir treatment over time in cCMV patients demonstrating that the change in viral load during antiviral therapy may not be useful for the prediction of hearing loss development in symptomatic cCMV patients.

Identification of the genetic cause of hearing loss has become common in Western countries. Notini et al. [5] present a collection of clinicians’ experiences and views with offering and returning results from exome sequencing to parents of infants with hearing loss. Whereas most clinicians are supportive of exome sequencing for children with hearing loss some of them doubt the utility of this information in case of isolated hearing loss. In addition, views on the optimal time to offer exome sequencing to families differs. This study certainly requires complementation by representative samples of parents directly reporting their views.

Three contributions relate to developmental assessments required for tailored early intervention. In accordance with the key role of parent-child interaction for child and family outcomes, Curtin et al. [6] present a systematic review on assessments of parent behaviors in parent-child interactions with deaf and hard of hearing infants. As a result, they present essential parent behaviors and methods of assessment that will be useful for the development of practicable measures that are required for the planning of family-centred intervention targets. Feasibility of a new measure to monitor the development of functional listening skills by parental report is demonstrated by Davis et al. [7]. Information from audiological assessments needs to be complemented by systematic observations on functional listening in everyday settings to inform parent behaviors and/or clinical decisions. Another study [8] investigates the validity of two well-established diagnostic instruments for autism spectrum disorders adapted for the use with deaf signing children. As a result, the combination of the adapted autism diagnostic interview (ADI-R) and direct assessment (autism diagnostic observation schedule version 2; ADOS-2) are recommended for clinical practice. This procedure can be regarded as a significant advancement in diagnosing children with hearing loss with additional disabilities. So far, common over- and underdiagnosis of autism in children with hearing loss has resulted in inappropriate intervention and unnecessary restrictions in group participation.

A number of contributions to this special issue relate directly to the practice and outcomes of family-centered early intervention. Dirks and Szarkowski [9], underline the relevance for early intervention providers to address the specific needs of fathers and mothers. Self-efficacy in fathers was shown to be related to higher involvement and perceived support from EI providers, emphasizing efforts to actively increase involvement of fathers. In a multicenter longitudinal North American study Wiggin et al. [10] investigated effects of increased frequency of early intervention sessions in children 9 to 36 months of age. By structural equation modeling controlling for maternal education, degree of hearing loss and meeting with the Joint committee on Infant Hearing 1-3-6 guidelines (completion of screening by 1 month, diagnosis of hearing loss by 3 months and enrolment in intervention by 6 months of age) significant effects of a higher number of intervention sessions at the first assessment on expressive vocabulary outcomes 9 months later was found. These results first time underline the effect of the frequency of early intervention. Even though Ching et al.’s paper [11] does not directly relate to early intervention we consider it as an important contribution to intervention practice in the first years of life. In a cross-sectional sample of 144 children with hearing loss at about age of 9 years they demonstrate that better pragmatic language skills and functional listening skills rather than structural language abilities (e.g., vocabulary or morphosyntax) are associated with better psychosocial abilities and quality of life. These relationships in school-age children underline the relevance of social communication and functional listening rather than a focus on teaching of vocabulary, grammar or training listening skills independent of everyday settings from very early on.

Finally, our Linz group [12] presents the design of a comprehensive longitudinal study including all children with permanent hearing loss from birth to the age of 6 years aiming for the identification of child-, family- and intervention-related predictors of developmental trajectories. First descriptive data show the high rates of children with additional disabilities (31%) and those growing up with a minority language (32%) that are usually excluded from longitudinal studies. In addition to the inclusive character the study is characterized by its multidimensionality investigating medical, audiological, communicative, cognitive and psychosocial dimensions of development and in particular factors that can be influenced by intervention (e.g., parent-child interaction, parental self-efficacy, parental stress, quality of fitting of hearing technology and age at first fitting, use of hearing technology). The study is designed to allow for data pooling with other longitudinal total population studies. Multidisciplinarity is another characteristic of the study including parent, medical, speech-language, audiological, psychological and interventionist involvement at all stages.

The international contributions to this special issue bring together the evidence that support the effectiveness of well-integrated multidisciplinary networks, including family-centeredness in early identification and parent counselling, developmental surveillance, implementation of early intervention and upcoming research. There is still a tremendous need to increase global contributions to the field of early intervention in pediatric hearing loss.
